# Suppression of AGO2 by miR-132 as a determinant of miRNA-mediated silencing in human primary endothelial cells

**DOI:** 10.1016/j.biocel.2015.10.006

**Published:** 2015-12

**Authors:** German Leonov, Kunal Shah, Daniel Yee, Jon Timmis, Tyson V. Sharp, Dimitris Lagos

**Affiliations:** a Centre for Immunology and Infection, Department of Biology and Hull York Medical School University of York, Wentworth Way, York YO10 5DD, UK; b Barts Cancer Institute, John Vane Science Centre, Charterhouse Square, Queen Mary University London, London EC1M 6BQ, UK; c Department of Electronics, Wentworth Way, York YO10 5DD, UK

**Keywords:** Lymphatic endothelial cells, microRNA, RNA-binding proteins, AGO2, miR-132

## Abstract

The abundance of miR-132 ranges from constitutively high in the brain where it is necessary for neuronal development and function, to inducible expression in haematopoietic and endothelial cells where it controls angiogenesis and immune activation. We show that expression of AGO2, a protein central to miRNA-mediated gene silencing and miRNA biogenesis, is negatively regulated by miR-132. Using HeLa cells, we demonstrate that miR-132 interacts with the AGO2 mRNA 3′UTR and suppresses AGO2 expression and AGO2-dependent small RNA-mediated silencing. Similarly, miR-132 over-expression leads to AGO2 suppression in primary human dermal lymphatic endothelial cells (HDLECs). During phorbol myristate acetate (PMA)-activation of HDLECs, miR-132 is induced in a CREB-dependent manner and inhibition of miR-132 results in increased AGO2 expression. In agreement with the role of AGO2 in maintenance of miRNA expression, AGO2 suppression by miR-132 affects the steady state levels of miR-221 and miR-146a, two miRNAs involved in angiogenesis and inflammation, respectively. Our data demonstrate that the miRNA-silencing machinery is subject to autoregulation during primary cell activation through direct suppression of AGO2 by miR-132.

## 1 Introduction

The mature miR-132 (miR-132-3p) is derived from the primary miR-132/miR-212 cluster, found in the intergenic region of chromosome 17p13.3. miR-132 and miR-212 have the same seed sequence and potential targets, although miR-132 is the preferentially expressed miRNA from the cluster (Lagos et al., 2010). The transcription of miR-132/miR-212 cluster is dependent on cAMP response element-binding (CREB) protein phosphorylation (Vo et al., 2005), which is inducible in an ERK-1/2 and MSK-1/2 dependent manner (Remenyi et al., 2010). miR-132 has been implicated in neuronal function and development (Remenyi et al., 2010, Wayman et al., 2008), circadian rhythm control (Alvarez-Saavedra et al., 2011), angiogenesis (Anand et al., 2010), and regulation of innate immune responses (Lagos et al., 2010, Shaked et al., 2009). In endothelial cells, upregulation of miR-132 positively controls proliferation, angiogenesis and tumour growth in response to vascular endothelial growth factor A (VEGF-A) by suppressing p120RasGap (RASA1) (Anand et al., 2010). During viral infection of human cells *in vitro*, miR-132 acts as a negative regulator of inflammation in endothelial cells and macrophages by modulating EP300 (Lagos et al., 2010). Interestingly, EP300 is a co-transcriptional activator that is required for miR-132 expression, which may contribute to the transient expression of this miRNA, as seen in endothelial cells. Although the expression of miR-132 is low under basal conditions in HDLECs (approximately 50–100 copies per cell), endothelial activation leads to an early upregulation of miR-132 required for endothelial cell proliferation and angiogenesis (Anand et al., 2010, Lagos et al., 2010).

The abundance of mature miRNAs can be regulated at multiple steps of miRNA biogenesis by altering the expression or function of miRNA machinery proteins. The knock-down of DICER (Zhang et al., 2012, Schmitter et al., 2006) or AGO2 (Schmitter et al., 2006) has been correlated with a global drop of mature miRNA expression levels. DICER (Bernstein et al., 2003) and AGO2 (Morita et al., 2007) knock-out mice are embryonic lethal and this demonstrates the importance of miRNA-mediated regulation of gene expression during the early stages of embryo development. Reversely, the upregulation of AGO2 has been associated with a global increased expression of miRNAs (Martinez and Gregory, 2013, Winter and Diederichs, 2011). Global changes in miRNA expression levels lead to disruptions in cellular function, such as poor neovascularisation and vascular deformation reported in *DICER* knock-out mice (Bernstein et al., 2003).

The very nature of miRNA-mediated gene regulation means that the formation of negative feedback regulatory loops can occur, where a miRNA can be involved in regulating its own biogenesis by targeting components required for its specific transcription, post-transcriptional processing, stability, or target accessibility. In the context of miRNA biogenesis, such regulatory loops have been identified in the regulation of DICER by *let-7* in mammalian cell lines (Tokumaru et al., 2008), AGO1 regulation by miR-168 in plants (Martínez de Alba et al., 2011), and ALG-1 regulation by *let-7* in *Caenorhabditis elegans* (Zisoulis et al., 2012). The regulation of mammalian AGO2 by miR-184 has also been reported during cytokine stimulation (Roberts et al., 2013), and reported in response to insulin stimulation and thereby controlling cell proliferation (Tattikota et al., 2014). Of note, AGO2 is the only catalytically active mammalian Argonaute family protein (Meister et al., 2004), making it essential for mRNA cleavage and effective in both siRNA and miRNA-mediated silencing.

Here, we identify an autoregulatory feedback mechanism that involves AGO2 suppression by miR-132. We have examined this mechanism in both transformed cell lines and activated primary human dermal lymphatic endothelial cells (HDLECs). We demonstrate that changes in AGO2 expression affect expression of specific HDLEC miRNAs, such as miR-221 and miR-146a, which have important and critical roles in angiogenesis and inflammation (Li et al., 2009, Poliseno et al., 2006, Taganov et al., 2006, Yang et al., 2012). Overall, our findings reveal a novel mechanism regulating AGO2 expression and provide mechanistic insight into the function of miR-132 in human primary endothelial cells.

## 2 Methods

### 2.1 Cell culture

Human dermal lymphatic endothelial cells (HDLEC) were purchased from Promocell and grown in endothelial cell growth medium (Promocell) supplemented with 10 ng/mL VEGF-C (R&D). All HDLEC experiments were performed at passage 5. HDLECs (passage 5) fully maintain their proliferative capacity and differentiation markers (Hansen et al., 2010, Lagos et al., 2010), including LYVE1 (Supplementary Fig. 1). HeLa cells were grown in Dulbecco's modified eagle medium (DMEM) containing 10% FCS.

### 2.2 Transfections with miRNA mimics and inhibitors, and RNA inhibitors

Cells were transfected in 6-well plates in Opti-MEM using Oligofectamine (Invitrogen) transfection reagent 18 h after seeding. The siRNAs (OnTargetPlus SmartPool from Thermo Scientific), miRNA mimics (miRIDIAN from Thermo Scientific) were prepared at 25 nM concentration and Locked Nucleic Acid (LNA)-inhibitors of miR-132 (Exiqon) were prepared at 50 nM concentration. Cells were harvested 48 h post transfection.

### 2.3 Lentiviral transduction

The miR-132/miR-212 cluster or AGO2-UTR were amplified from genomic DNA or cDNA respectively and subcloned into the pSIN lentiviral vector using the NotI and BamHI restriction enzymes. For lentiviral transduction, virions were produced as described in (Lagos et al., 2008). HDLECs were infected for 48 h before harvesting.

### 2.4 PMA treatment

HDLECs were activated with PMA for 24 h before harvesting. PMA treatments were carried out 18 h after seeding cells into 6-well plates, 48 h after miRNA inhibition, mimics transfection or siRNA transfection, and 30 h after lentiviral transduction.

### 2.5 RNA preparation and qRT-PCR

Total RNA was extracted using the miRNEasy kit (Qiagen). Levels of mRNA were quantified by qRT-PCR using the TaqMan Universal PCR Master Mix (Applied Biosystems) for AGO2 and pri-miR-132 (TaqMan primers from Applied Biosystems) and using the SYBR Green Master Mix (Applied Biosystems) for *GAPDH* (forward: 5′-GGAGTCAACGGATTTGGTCGTA-3′; reverse: 5′-GGCAACAATATCCACTTTACCAGAGT-3′) and pri-miR-126 (forward: 5′-TATCAGCCAAGAAGGCAGAA-3′, reverse: 5′-CGTGGCGTCTTCCAGAAT-3′). Primers were used at 300 nM final concentration. *GAPDH* was used as a loading control for AGO2, pri-miR-126 and pri-miR-132. *RNU6* was used as a loading control for all mature miRNAs (Applied Biosystems).

### 2.6 Immunoblots and antibodies

Primary antibodies were prepared at 1:1000 for AGO1 (Cell Signalling, D84G10), AGO2 (Cell Signalling, C34C6), CREB (Cell Signalling, 48H2), phospho-CREB at Ser133 (Cell Signalling, 87G3), 1:500 for EP300 (Abcam, 3G230), RASA1 (Santa Cruz, B4F8), and 1:5000 for β-actin (Cell Signalling, AC-15). Secondary goat anti-mouse (Dako, P0447) and goat anti-rabbit (Dako, P0448) antibodies were prepared at 1:5000 conjugated to HRP. Western blot analysis was carried out using ImageJ software for quantifying band intensities.

### 2.7 Luciferase reporter assay

AGO2 3′UTR and EP300 3′UTR were amplified from HDLEC cDNA and subcloned into the psiCheck2 vector (Promega) using *Not*I and *Xho*I enzymes. Mutations were introduced at the AGO2 3′UTR at the miR-132 binding site (WT: 5′-GUACAAUCCUUUUUCACUGUUU-3′; Mut: 5′-GUACAAUCCUUUUUCACUAAAU-3′). Luciferase assays were performed in HeLa cells. AGO2 and EP300 UTR constructs were transfected for 24 h after the 24 h miR-132 mimics (20 nM) or NTC transfection. Relative light units (RLU) were measured with a Fluoroscan Ascent FL luminometer (Thermo Scientific).

### 2.8 Let-7 silencing reporter assay

The *let-7* silencing reporter assay was carried out in HeLa cells as previously described by (James et al., 2010) in the presence of NTC/miR-132 mimics (20 nM) or siNTC/siAGO2 (20 nM). The luciferase reporter used (40 nM) contained either a single fully complementary site (si) or a seven times repeat of a mismatched site (mi7) for *let-7* binding.

### 2.9 Statistical analysis

Experimental results are presented as mean ± S.E. (*error bars*) and were performed in 3 independent replicates unless otherwise stated in the figure legend. Where appropriate, a two-tailed unpaired Student's *t*-test was used to calculate the significance of the difference between treatments.

## 3 Results

### 3.1 miR-132 interacts with the AGO2 3′UTR

**Fig. 1:**
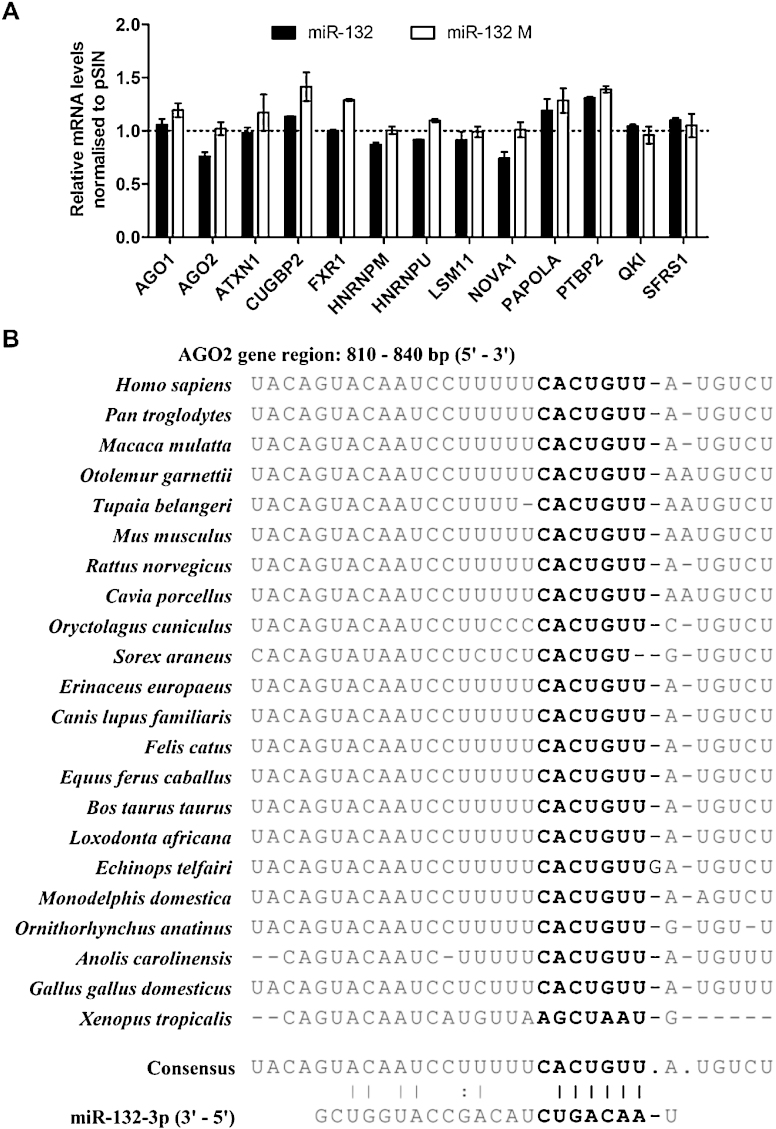
miR-132 interacts with AGO2 3′UTR. (A) mRNA levels of RBPs following lentiviral overexpression of miR-132 and miR-132 mutant (M) in HDLECs, 48 h post-transduction (average mRNA levels from two independent screens shown). (B) Conservation of AGO2 3′UTR region targeted by miR-132 (bold font – ‘seed’ site of miR-132 binding on AGO2 3′UTR).

Gene ontology analysis of predicted miR-132 targets, using the EIMMO prediction analysis tool (Hausser et al., 2009), indicated that RNA-binding proteins (RBPs) were over-represented amongst miR-132 predicted targets (Supplementary Table 1). We utilised a lentiviral construct containing the miR-132/miR-212 locus (Lagos et al., 2010) to screen for the effect of miR-132 on mRNA levels of 13 RBPs that were predicted miR-132 targets by multiple algorithms (Supplementary Table 2). Over-expression of miR-132 and miR-212 in HDLECs resulted in a drop in AGO2 mRNA (Fig. 1A). The effect was abolished when using a construct where the seed sequence of both miR-132 and miR-212 is mutated (Fig. 1A).

**Fig. 2:**
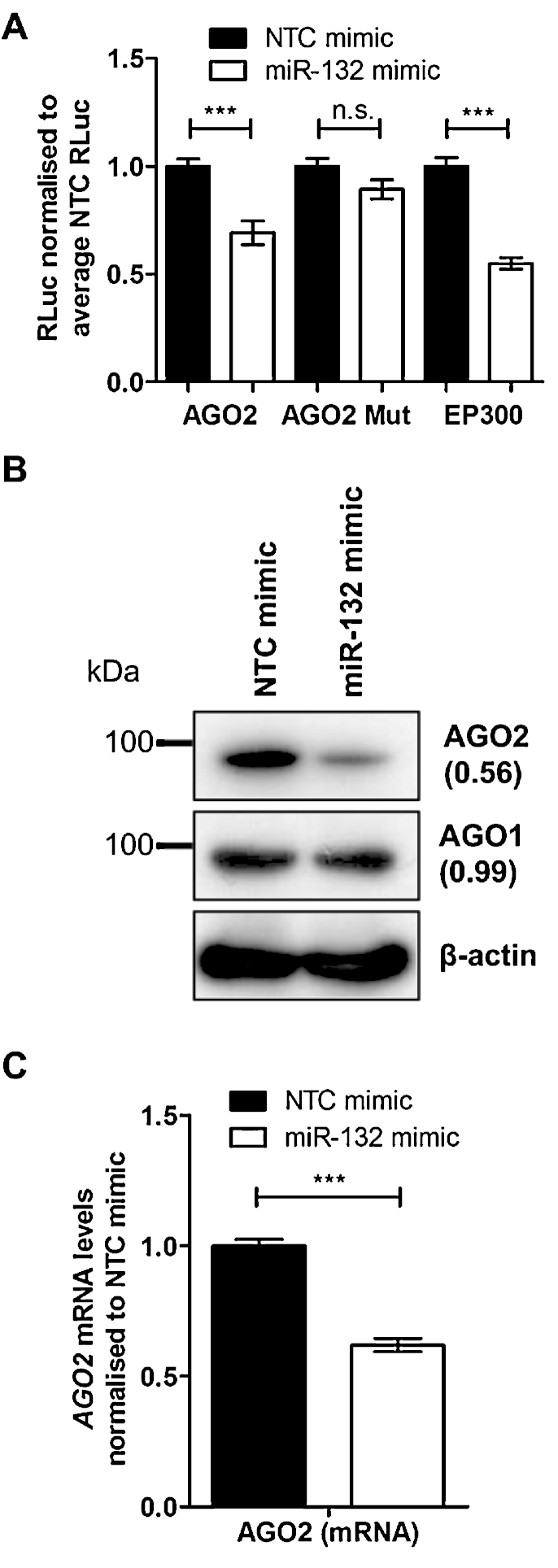
miR-132 over-expression suppresses AGO2 expression in HeLas. (A) Relative Renilla luciferase (Rluc) activity for AGO2 WT 3′UTR, AGO2 Mutant 3′UTR and EP300 WT 3′UTR, normalised to the average Rluc activity of each individual replicate experiment in HeLas. (B) Expression of AGO1 and AGO2 protein in HeLas, 48 h post-transfection with miR-132 mimics. (C) Levels of *AGO2* mRNA in HeLas, 48 h post-transfection with miR-132 mimics. *** Indicates *p* < 0.001.

We identified a potential miR-132 binding site in the AGO2 3′UTR. The binding site is conserved across mammalian species with the exception of *Sorex araneus* (Fig. 1B). Based on this, we further investigated whether miR-132 interacted with the AGO2 3′UTR region. The introduction of miR-132 mimic into HeLa cells led to a drop in luciferase signal for a reporter containing the AGO2 3′UTR (Fig. 2A), compared to the non-targeting control (NTC). A triple consecutive base pair mutation introduced at the start of the seed site of the miR-132 binding in the AGO2 3′UTR reversed the suppression of luciferase activity by miR-132 mimic. The effect of miR-132 on the AGO2 3′UTR was similar to that on the EP300 3′UTR, a known miR-132 target (Lagos et al., 2010). We should note that endogenous miR-132 expression in HeLa cells is at similarly low levels to those observed in HDLEC.

### 3.2 miR-132 over-expression suppresses AGO2 expression

**Fig. 3:**
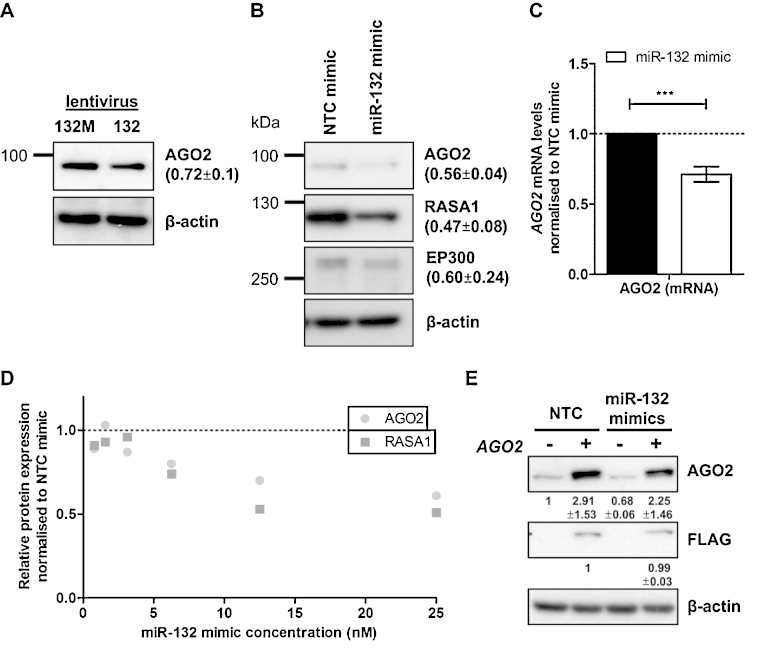
miR-132 over-expression suppresses AGO2 expression in HDLECs. (A) Expression of AGO2 protein following lentiviral overexpression of miR-132 and miR-132 M in HDLECs, 48 h post-transduction. (B) Expression of AGO2, RASA1, EP300 protein in HDLECs, 48 h post-transfection with miR-132 mimics. (C) mRNA levels of *AGO2* in HDLECs, 48 h post-transfection with miR-132 mimics. *** Indicates *p* < 0.001. (D) Expression of AGO2 and RASA1 protein after a titration of miR-132 mimics in HDLECs, 48 h post-transfection. (E) AGO2 protein expression of two experiments with a 24 h lentiviral overexpression of AGO2^−UTR^ following a miR-132 overexpression in HDLECs.

Having shown that miR-132 can directly interact with the predicted miR-132-binding site in the AGO2 3′UTR, we studied the effect of miR-132 over-expression on AGO2 expression. Transfection of miR-132 synthetic mimics into HeLa cells resulted in a decrease in AGO2 protein and mRNA levels (Fig. 2B and C). Similarly, in HDLECs the observed decrease in AGO2 mRNA levels following over-expression of the miR-132/miR-212 cluster (Fig. 1A) correlated with decreased AGO2 protein levels (Fig. 3A). Transfection of synthetic miR-132 mimics into HDLECs suppressed AGO2 protein expression to the same extent as the known miR-132 targets EP300 and RASA1 (Fig. 3B), and also resulted in a drop in AGO2 mRNA (Fig. 3C). The suppression of AGO2 in HDLECs was miR-132-dose-dependent with maximum suppression observed when using 15–25 nM concentration (Fig. 3D; Supplementary Fig. 2). To verify that the effect of miR-132 on AGO2 expression in HDLECs was indeed through direct targeting of the AGO2 3′UTR region, we utilised a lentiviral construct expressing FLAG-tagged AGO2 but lacking its 3′UTR region (AGO2^−UTR^). We found that AGO2^−UTR^ expression in HDLECs was not affected by miR-132 mimics (indicated by anti-FLAG M2, Fig. 3E). As expected, miR-132 mimics in AGO2^−UTR^ over-expressing HDLECs resulted in a drop in endogenous AGO2, shown by a decrease in total AGO2 levels.

### 3.3 miR-132 over-expression affects AGO2 function

**Fig. 4:**
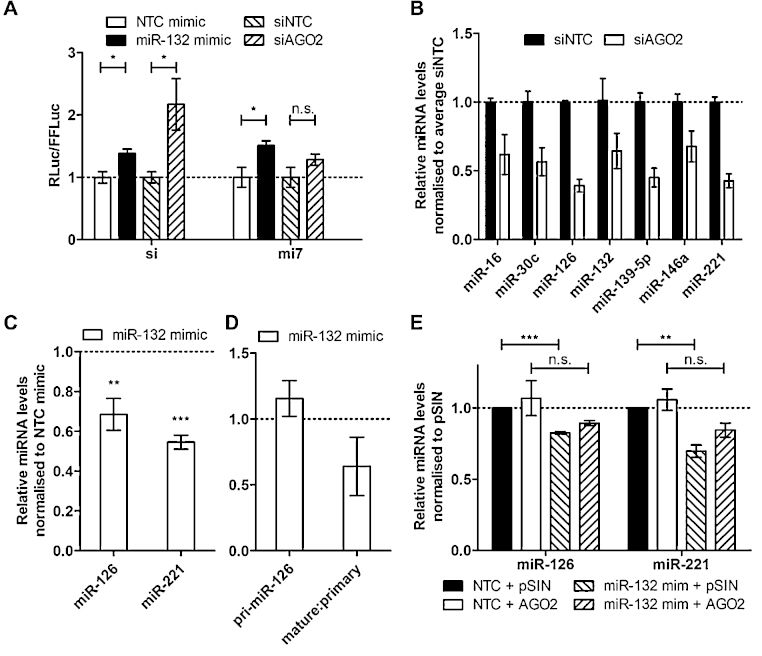
Over-expression of miR-132 affects AGO2 function in HDLECs. (A) *Let-7* silencing activity reporter assay with a fully complementary (si) and multiple mismatched (mi7) *let-7* binding sites (16) in HeLas. (B) Effect of AGO2 knockdown on miR-16, miR-30c, miR-126, miR-132, miR-139-5p, miR-146a and miR-221 levels in HDLECs, 48 h post-transfection with siAGO2 (*n* = 2). (C) Levels of miR-126 and miR-221 in HDLECs, 48 h post-transfection with miR-132 mimics. D. Levels of primary miR-126 and mature to primary miR-126 in HDLECs, 48 h post-transfection with miR-132 mimics. (E) miR-126 and miR-221 levels of two experiments with a 24 h lentiviral overexpression of AGO2^−UTR^ following a miR-132 overexpression in HDLECs. * Indicates *p* < 0.05, ** indicates *p* < 0.01, and *** indicates *p* < 0.001, n.s. not significant.

Next, we tested whether the observed level of AGO2 regulation by miR-132 was functionally relevant. We employed an endogenous let-7a silencing reporter constructs containing a fully complementary (si) or multiple copies of bulged (mi7) Let-7 binding sites downstream of a luciferase reporter as previously described (James et al., 2010). The si reporter gives a read-out of the RNAi function of AGO2, which relies on full complementarity between the small RNA (in this case let-7a) and the target. The mi7 reporter provides a read-out of the miRNA silencing function of Ago2 as it is not susceptible to cleavage by AGO2 endonuclease activity. We observed that the introduction of miR-132 mimics or siAGO2 resulted in derepression of both siRNA and miRNA reporter constructs, as indicated by derepression of the Rluc activity (Fig. 4A). These findings show that miR-132-mediated regulation of AGO2 is sufficient to negatively impact AGO2 siRNA and miRNA-mediated silencing functions, thus further defining the importance of this regulatory feedback mechanism.

To test the functional significance of miR-132-mediated suppression of AGO2 in HDLECs, we took advantage of the fact that loss of AGO2 expression results in decreased miRNA expression (Schmitter et al., 2006, Morita et al., 2007). We confirmed that this was the case in HDLECs by determining expression of several miRNAs following siRNA mediated knockdown of AGO2 (Fig. 4B). Similarly, miR-132-mediated AGO2 suppression resulted in down-regulation of miR-126 and miR-221 (Fig. 4C). The decrease in mature miR-126 levels was due to a reduction in the mature to primary miRNA ratio (Fig. 4D), suggesting that post-transcriptional regulation of miR-126 contributes to the reduction in its levels in miR-132-over-expressing HDLECs, being consistent with the effect of miR-132 on AGO2 expression. This was further supported by the fact that physiologically relevant over-expression of AGO2^−UTR^ in HDLECs transfected with control or miR-132 mimics partially restored miR-126 and miR-221 expression. Over-expression of miR-132 in HDLECs transduced with control lentivirus (pSIN) resulted in statistically significant down-regulation of miR-126 and miR-221. In contrast, transfection of miR-132 mimics in HDLECs transduced with AGO2 lentivirus did not significantly affect miR-126 and miR-221 levels (Fig. 4E).

### 3.4 PMA induces miR-132 expression in HDLECs

**Fig. 5:**
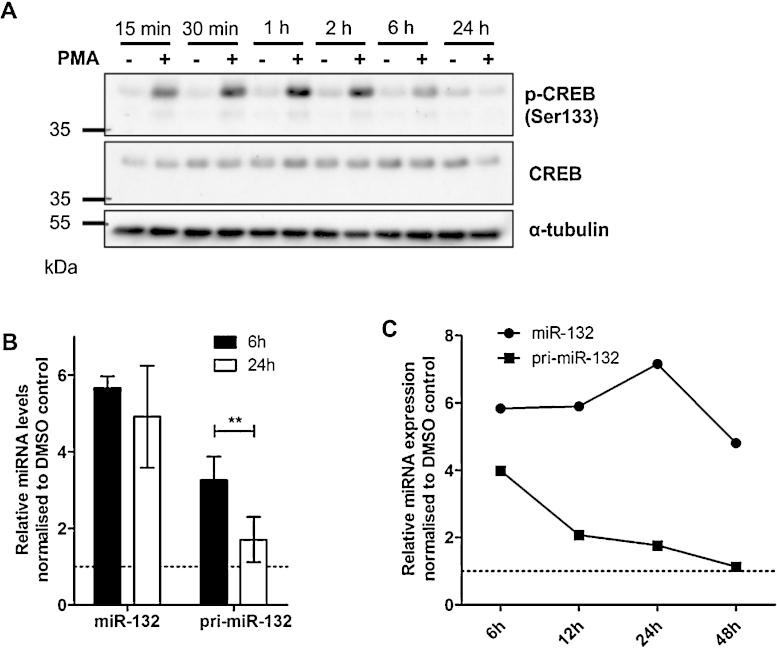
PMA induces miR-132 expression by activating CREB. (A) Expression of phospho-CREB during PMA activation (25 nM) in HDLECs over a 24 h period. (B) Levels of mature and primary miR-132 in HDLECs, 6 h and 24 h post-PMA treatment (*n* = 4). ** Indicates *p* < 0.01. (C) Time course of mature and primary miR-132 levels in HDLECs treated with PMA over a 48 h period.

miR-132 expression is transcriptionally controlled by CREB activation (Lagos et al., 2010, Remenyi et al., 2010, Vo et al., 2005). We used PMA, a potent activator of PKC that results in CREB phosphorylation (Mao et al., 2007). We showed that CREB is phosphorylated during the first few hours post-PMA treatment and the effect is lost after 24 h (Fig. 5A; Supplementary Fig. 3A). The activation of CREB led to an induction of mature miR-132 expression over a range of PMA concentrations (Supplementary Fig. 3B). The most potent and sustained induction of miR-132 was obtained following treatment with 25 nM PMA and this concentration was used for all further experiments.

The expression of primary and mature miR-132 was measured at 6 h and 24 h post-PMA treatment (Fig. 5B) and a more detailed time-course of expression was performed over 48 h (Fig. 5C). Consistent with CREB-mediated transcriptional activation, pri-miR-132 was up regulated during the first few hours post-PMA treatment and returned to near-baseline expression level by 24 h. However, mature miR-132 levels showed only a modest decrease over the time-course following the initial induction, suggesting it was more stable than pri-miR-132 in these conditions.

### 3.5 Inhibition of endogenous miR-132 leads to increased AGO2 expression

**Fig. 6:**
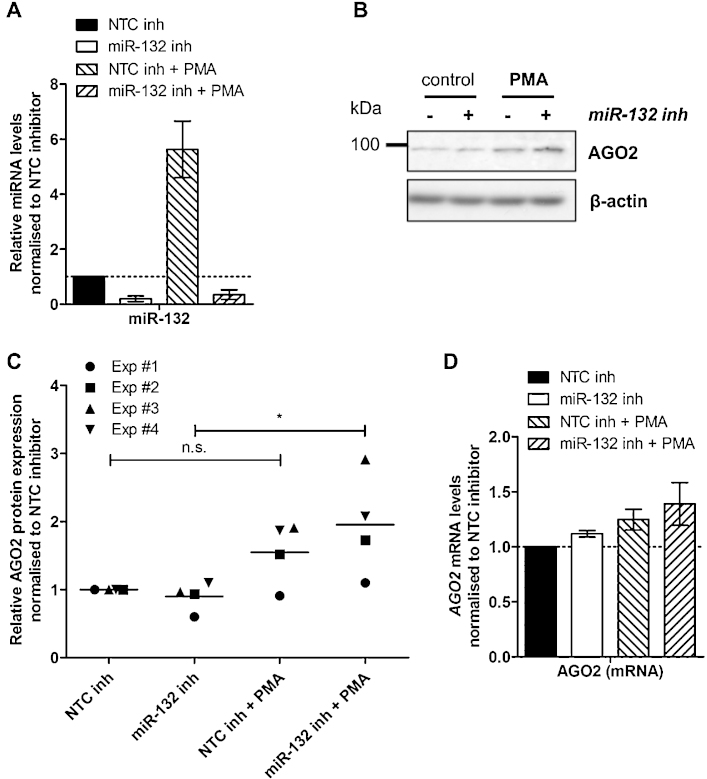
Inhibition of endogenous miR-132 leads to increased AGO2 expression. (A) Levels of miR-132 in HDLECs 24 h post-PMA treatment following miR-132 inhibition. (B) AGO2 protein expression in HDLECs 24 h post-PMA treatment following miR-132 inhibition. (C) Quantification of AGO2 protein expression 24 h post-PMA treatment following miR-132 inhibition in HDLECs, displaying the breakdown of the response in each individual experiment (*n* = 4). * Indicates *p* < 0.05, n.s. not significant. (D) Levels of *AGO2* mRNA 24 h post-PMA treatment following miR-132 inhibition in HDLECs (*n* = 4).

We have previously shown that miR-132 expression in HDLECs becomes functionally relevant only when induced (Lagos et al., 2010). Therefore, we exploited the PMA-inducible miR-132 expression *in vitro* system in HDLECs to determine the effect of endogenous miR-132 on AGO2 expression. Inhibition of miR-132 induction (Fig. 6A) resulted in statistically significant induction of AGO2 expression in PMA-treated HDLECs (24 h), an effect that was not observed in HDLECs transfected with control miRNA inhibitors (Fig. 6B and C). PMA treatment or miR-132 inhibition did not affect AGO2 mRNA levels (Fig. 6D).

**Fig. 7:**
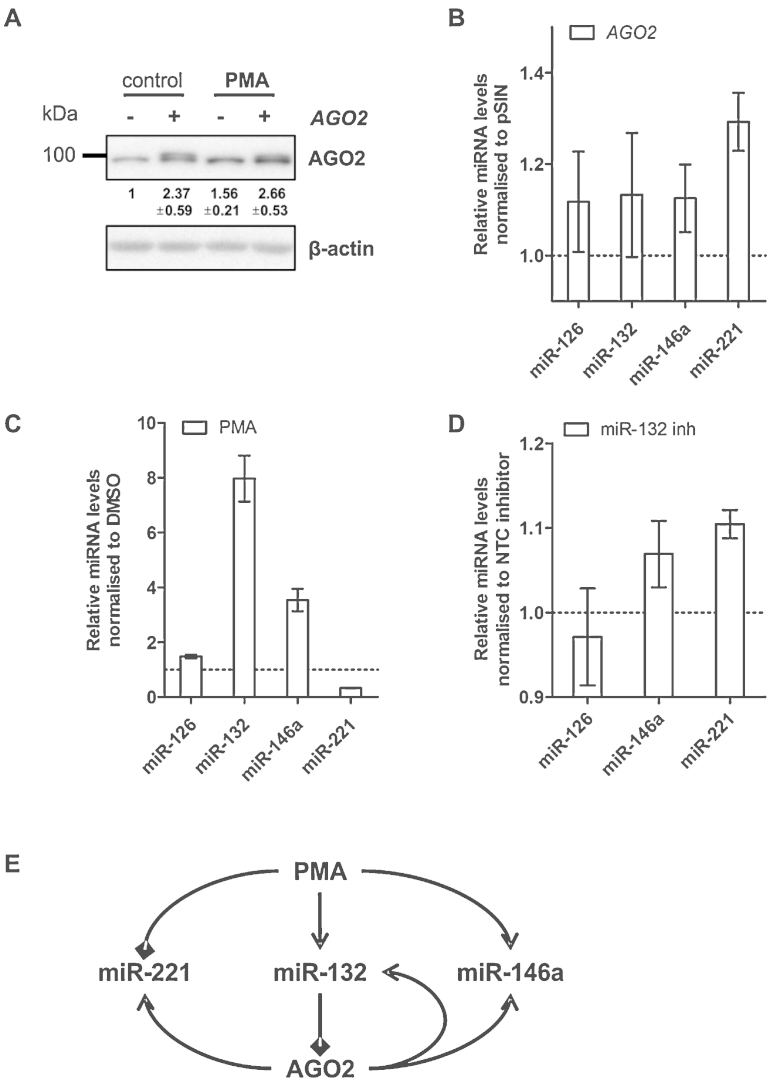
Regulation of AGO2 affects miRNA abundance. (A) AGO2 protein expression in HDLECs 24 h post-PMA treatment following a 30 h AGO2^−UTR^ lentiviral overexpression. (B) miR-126, miR-132, miR-146a and miR-221 levels in HDLECs 48 h post-AGO2^−UTR^ lentiviral overexpression. (C) miR-126, miR-132, miR-146a and miR-221 levels in HDLECs 24 h post-PMA treatment. (D) miR-126, miR-146a and miR-221 levels 48 h post-miR-132 LNA inhibition in PMA activated HDLECs. (E) Schematic diagram depicting PMA/miR-132/AGO2-associated regulatory loops: AGO2 regulation by a (1) negative feedback loop (miR-132), (2) coherent feed forward loop (miR-221), and (3) incoherent feed forward loop (miR-146a).

As the changes in AGO2 expression following PMA treatment and miR-132 inhibition were modest, we tested whether such effects could affect AGO2 function. We over-expressed AGO2^−UTR^ during PMA-activation of HDLECs to a similar level (Fig. 7A) as observed during miR-132 inhibition. This level of over-expression of AGO2^−UTR^ in HDLECs resulted in a modest increase in expression of miR-126, miR-132, miR-146a, and miR-221, the effect being more prominent for the latter two miRNAs (Fig. 7B). miR-221 was down-regulated and miR-146a up-regulated during PMA-activation (Fig. 7C). In agreement with these findings and the effect of miR-132 on AGO2 expression (Fig. 6B), we observed an increase in miR-221 and miR-146a expression in response to miR-132 inhibition in PMA-activated HDLECs (Fig. 7D), while miR-126 expression was unaffected. Overall, these results further supported that endogenous miR-132 can regulate AGO2 expression in activated HDLECs.

## 4 Discussion

Here we demonstrate that miR-132 can suppress expression of AGO2 through a canonical miRNA-mediated silencing mechanism, and that this interaction fine-tunes AGO2 expression in activated primary endothelial cells. Our findings indicate that steady state expression of some miRNAs is highly sensitive to changes in AGO2 expression in HDLECs. This is in agreement with previous reports demonstrating that loss of AGO2 has variable effects on steady-state levels of individual miRNAs (Diederichs and Haber, 2007, Winter and Diederichs, 2011, Bronevetsky et al., 2013). Importantly, we find that miR-132 is involved in modulating the availability of AGO2 to perform its function in siRNA and miRNA-mediated silencing and in stabilising miRNA expression (Winter and Diederichs, 2011). This provides support for a model according to which post-transcriptional regulation of miR-132 by AGO2 is coupled to miR-132 mediated regulation of AGO2, maintaining optimal miR-132 and AGO2 expression during haematopoietic or endothelial cell activation. Although basal miR-132 expression is low in non-neuronal cells, during cellular activation its expression increases to functionally relevant levels as demonstrated by its role in modulating the innate immune response (Lagos et al., 2010) and angiogenesis (Anand et al., 2010). Of note, miR-132 has been previously shown to participate in similar negative feedback loops maintaining homeostatic levels of EP300 in endothelial and immune cells (Lagos et al., 2010) and MeCP2 expression in neurons, where the baseline expression of miR-132 is higher but also inducible (Klein et al., 2007). We speculate that the propensity of miR-132 to form negative feedback loops with its mRNA targets is essential for its activity-dependent expression and function both outside and within the brain. Our findings indicate that in PMA-treated HDLECs mature miR-132 is substantially longer-lived than its primary transcript, which might provide one possible explanation for the need for multiple negative feedback loops controlling miR-132 expression and function.

In our study, we focussed on endothelial cells as miR-132 has been shown to regulate angiogenic and inflammatory responses in these cells. For the same reason, when looking for functional consequences of AGO2 regulation we chose to measure expression of miRNAs that have previously been reported to be involved in angiogenesis or inflammation. For example, miR-221 (Poliseno et al., 2006, Li et al., 2009) is known as an anti-angiogenic miRNA, miR-126 is essential for endothelial cell survival (Sun et al., 2010, Wang et al., 2008), and miR-146a involved in inflammatory responses in endothelial cells (Lagos et al., 2010). We show that treatment of HDLECs with PMA results in an induction of the pro-angiogenic miR-132, and a decrease in the anti-angiogenic miR-221 expression, in agreement with the previously reported pro-angiogenic activity of PMA (Montesano and Orci, 1985). Interestingly, during endothelial cell activation, minimal over-expression of AGO2 or inhibition of miR-132 result in increased miR-221 and miR-146a expression, in agreement with the proposed miR-132-mediated AGO2 regulation. We should note that the magnitude of the effect of miR-132 inhibition on miR-221 and miR-146a indicates that other factors also contribute to the regulation of these miRNAs in activated HDLECs. However, our results suggest that regulation of AGO2 due to miR-132 induction is a failsafe mechanism that ensures maintained suppression of miR-221, and limits the extent of the anti-inflammatory activity miR-146a (Fig. 7E). Indeed, miRNAs are often components of such coherent (e.g. miR-221) or incoherent (e.g. miR-146a) feed forward loops (Herranz and Cohen, 2010). It would be interesting to further investigate the relevance of this intricate miRNA network in *in vivo* models of angiogenesis and in cells with high basal miR-132 expression, such as neurons.

Validated miR-132 targets are involved in chromatin modifications (e.g. MeCP2, EP300, Jarid1a, SIRT1) (Alvarez-Saavedra et al., 2011, Yamakuchi, 2012) transcription (e.g. EP300) (Hasan et al., 2001), and mRNA splicing (e.g. PTBP2) (Smith et al., 2011), indicating a functional clustering of miR-132 targets around modulators of the lifecycle of a mammalian mRNA. We substantially expand this concept to cover mRNA silencing, by revealing that miR-132 can suppress AGO2. The major implication of these findings is that a miR-132 can potentially act as a concurrent regulator of transcription, splicing, and silencing in the cell. Defining how these fundamental processes communicate is of paramount importance and particularly relevant to cellular stress responses, which are characterised by highly dynamic, and accurate gene expression programmes. We propose that the potential of miR-132 in coordinating multiple molecular processes that determine gene expression can provide crucial clues towards understanding the complex and context-specific role of this miRNA in neurons and haematopoietic and vascular cells.

## References

[bib0005] Alvarez-Saavedra M., Antoun G., Yanagiya A., Oliva-Hernandez R., Cornejo-Palma D., Perez-Iratxeta C., Sonenberg N., Cheng H.-Y.M. (2011). miRNA-132 orchestrates chromatin remodeling and translational control of the circadian clock. Hum. Mol. Genet..

[bib0010] Anand S., Majeti B.K., Acevedo L.M., Murphy E.A., Mukthavaram R., Scheppke L., Huang M., Shields D.J., Lindquist J.N., Lapinski P.E., King P.D., Weis S.M., Cheresh D.A. (2010). Micro RNA-132-mediated loss of p120RasGAP activates the endothelium to facilitate pathological angiogenesis. Nat. Med..

[bib0015] Bernstein E., Kim S.Y., Carmell M.A., Murchison E.P., Alcorn H., Li M.Z., Mills A.A., Elledge S.J., Anderson K.V., Hannon G.J. (2003). Dicer is essential for mouse development. Nat. Genet..

[bib0020] Bronevetsky Y., Villarino A., Eisley V.C., Barbeau J., Barczak R.A., Heinz J.G., Kremmer A., Heissmeyer E., McManus V.M., Erle T.D., Rao J., Ansel A.K.M. (2013). T cell activation induces proteasomal degradation of Argonaute and rapid remodeling of the microRNA repertoire. J. Exp. Med..

[bib0025] Diederichs S., Haber D.A. (2007). Dual role for argonautes in microRNA processing and posttranscriptional regulation of microRNA expression. Cell.

[bib0030] Hansen A., Henderson S., Lagos D., Nikitenko L., Coulter E., Roberts S., Gratrix F., Plaisance K., Renne R., Bower M., Kellam P., Boshoff C. (2010). KSHV-encoded miRNAs target MAF to induce endothelial cell reprogramming. Genes Dev..

[bib0035] Hasan S., Hassa P., Imhof O., Hottiger R.M.O. (2001). Transcription coactivator p300 binds PCNA and may have a role in DNA repair synthesis. Nature.

[bib0040] Hausser J., Berninger P., Rodak C., Jantscher Y., Wirth S., Zavolan M. (2009). MirZ: an integrated microRNA expression atlas and target prediction resource. Nucleic Acids Res..

[bib0045] Herranz H., Cohen S.M. (2010). MicroRNAs and gene regulatory networks: managing the impact of noise in biological systems. Genes Dev..

[bib0050] James V., Zhang Y., Foxler D.E., de Moor C.H., Kong Y.W., Webb T.M., Self T.J., Feng Y., Lagos D., Chu C.-Y., Rana T.M., Morley S.J., Longmore G.D., Bushell M., Sharp T.V. (2010). LIM-domain proteins, LIMD1, Ajuba, and WTIP are required for microRNA-mediated gene silencing. Proc. Natl. Acad. Sci. U. S. A..

[bib0055] Klein M., Lioy E.D., Ma T., Impey L., Mandel S., Goodman G.R.H. (2007). Homeostatic regulation of MeCP2 expression by a CREB-induced microRNA. Nat. Neurosci..

[bib0060] Lagos D., Vart R.J., Gratrix F., Westrop S.J., Emuss V., Wong P.-P., Robey R., Imami N., Bower M., Gotch F., Boshoff C. (2008). Toll-like receptor 4 mediates innate immunity to Kaposi sarcoma herpesvirus. Cell Host Microbe.

[bib0065] Lagos D., Pollara G., Henderson S., Gratrix F., Fabani M., Milne R.S.B., Gotch F., Boshoff C. (2010). miR-132 regulates antiviral innate immunity through suppression of the p300 transcriptional co-activator. Nat. Cell Biol..

[bib0070] Li Y., Song Y.-H., Li F., Yang T., Lu Y.W., Geng Y.-J. (2009). MicroRNA-221 regulates high glucose-induced endothelial dysfunction. Biochem. Biophys. Res. Commun..

[bib0075] Mao L., Tang Q., Wang J.Q. (2007). Protein kinase C-regulated cAMP response element-binding protein phosphorylation in cultured rat striatal neurons. Brain Res. Bull..

[bib0080] Martínez de Alba A.E., Jauvion V., Mallory A.C., Bouteiller N., Vaucheret H. (2011). The miRNA pathway limits AGO1 availability during siRNA-mediated PTGS defense against exogenous RNA. Nucleic Acids Res..

[bib0085] Martinez N.J., Gregory R.I. (2013). Argonaute2 expression is post-transcriptionally coupled to microRNA abundance. RNA.

[bib0090] Meister G., Landthaler M., Patkaniowska A., Dorsett Y., Teng G., Tuschl T. (2004). Human Argonaute2 mediates RNA cleavage targeted by miRNAs and siRNAs. Mol. Cell.

[bib0095] Montesano R., Orci L. (1985). Tumor-promoting phorbol esters induce angiogenesis in vitro. Cell.

[bib0100] Morita S., Horii T., Kimura M., Goto Y., Ochiya T., Hatada I. (2007). One Argonaute family member, Eif2c2 (Ago2), is essential for development and appears not to be involved in DNA methylation. Genomics.

[bib0105] Poliseno L., Tuccoli A., Mariani L., Evangelista M., Citti L., Woods K., Mercatanti A., Hammond S., Rainaldi G. (2006). MicroRNAs modulate the angiogenic properties of HUVECs. Blood.

[bib0110] Remenyi J., Hunter C.J., Cole C., Ando H., Impey S., Monk C.E., Martin K.J., Barton G.J., Hutvagner G., Arthur J.S.C. (2010). Regulation of the miR-212/132 locus by MSK1 and CREB in response to neurotrophins. Biochem. J..

[bib0115] Roberts J.C., Warren R.B., Griffiths C.E.M., Ross K. (2013). Expression of microRNA-184 in keratinocytes represses argonaute 2. J. Cell. Physiol..

[bib0120] Schmitter D., Filkowski J., Sewer A., Pillai R.S., Oakeley E.J., Zavolan M., Svoboda P., Filipowicz W. (2006). Effects of Dicer and Argonaute down-regulation on mRNA levels in human HEK293 cells. Nucleic Acids Res..

[bib0125] Shaked I., Meerson A., Wolf Y., Avni R., Greenberg D., Gilboa-Geffen A., Soreq H. (2009). MicroRNA-132 potentiates cholinergic anti-inflammatory signaling by targeting acetylcholinesterase. Immunity.

[bib0130] Smith P.Y., Delay C., Girard J., Papon M.A., Planel E., Sergeant N., Buée L., Hébert S.S. (2011). MicroRNA-132 loss is associated with tau exon 10 inclusion in progressive supranuclear palsy. Hum. Mol. Genet..

[bib0135] Sun Y., Bai Y., Zhang F., Wang Y., Guo Y., Guo L. (2010). miR-126 inhibits non-small cell lung cancer cells proliferation by targeting EGFL7. Biochem. Biophys. Res. Commun..

[bib0140] Taganov K., Boldin D.M., Chang P.K., Baltimore J.D. (2006). NF-kappaB-dependent induction of microRNA miR-146, an inhibitor targeted to signaling proteins of innate immune responses. Proc. Natl. Acad. Sci. U. S. A..

[bib0145] Tattikota S.G., Rathjen T., McAnulty S.J., Wessels H.-H., Akerman I., van de Bunt M., Hausser J., Esguerra J.L.S., Musahl A., Pandey A.K., You X., Chen W., Herrera P.L., Johnson P.R., O’Carroll D., Eliasson L., Zavolan M., Gloyn A.L., Ferrer J., Shalom-Feuerstein R., Aberdam D., Poy M.N. (2014). Argonaute2 mediates compensatory expansion of the pancreatic β cell. Cell Metab..

[bib0150] Tokumaru S., Suzuki M., Yamada H., Nagino M., Takahashi T. (2008). let-7 regulates Dicer expression and constitutes a negative feedback loop. Carcinogenesis.

[bib0155] Vo N., Klein M.E., Varlamova O., Keller D.M., Yamamoto T., Goodman R.H., Impey S. (2005). A cAMP-response element binding protein-induced microRNA regulates neuronal morphogenesis. Proc. Natl. Acad. Sci. U. S. A..

[bib0160] Wang S., Aurora A.B., Johnson B.A., Qi X., McAnally J., Hill J.A., Richardson J.A., Bassel-Duby R., Olson E.N. (2008). The endothelial-specific microRNA miR-126 governs vascular integrity and angiogenesis. Dev. Cell.

[bib0165] Wayman G.A., Davare M., Ando H., Fortin D., Varlamova O., Cheng H.-Y.M., Marks D., Obrietan K., Soderling T.R., Goodman R.H., Impey S. (2008). An activity-regulated microRNA controls dendritic plasticity by down-regulating p250GAP. Proc. Natl. Acad. Sci. U. S. A..

[bib0170] Winter J., Diederichs S. (2011). Argonaute proteins regulate microRNA stability: Increased microRNA abundance by Argonaute proteins is due to microRNA stabilization. RNA Biol..

[bib0175] Yamakuchi M. (2012). MicroRNA regulation of SIRT1. Front. Physiol..

[bib0180] Yang L., Boldin M., Yu P., Liu Y.C., Ea S.C., Ramakrishnan K., Taganov P.K., Zhao D.J., Baltimore L.D. (2012). miR-146a controls the resolution of T cell responses in mice. J. Exp. Med..

[bib0185] Zhang C., Huys A., Thibault P.A., Wilson J.A. (2012). Requirements for human Dicer and TRBP in microRNA-122 regulation of HCV translation and RNA abundance. Virology.

[bib0190] Zisoulis D.G., Kai Z.S., Chang R.K., Pasquinelli A.E. (2012). Autoregulation of microRNA biogenesis by let-7 and Argonaute. Nature.

