# Supplementary figures and images for: Suppression of AGO2 by miR-132 as a determinant of miRNA-mediated silencing in human primary endothelial cells
Source: Int J Biochem Cell Biol. 2015 Dec;69:75–84. doi: 10.1016/j.biocel.2015.10.006 (PMC4679077; doi:10.1016/j.biocel.2015.10.006)

**Supplementary Figure 1**

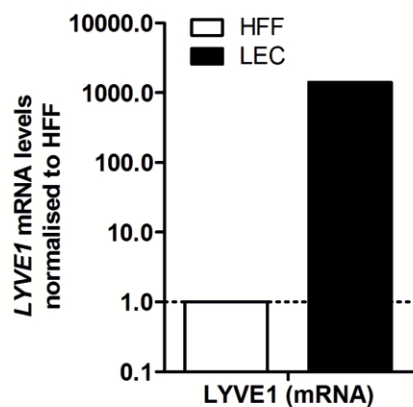

**Supplementary Figure 2**

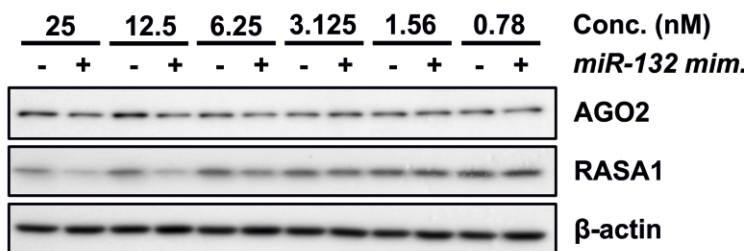

**Supplementary Figure 3A**

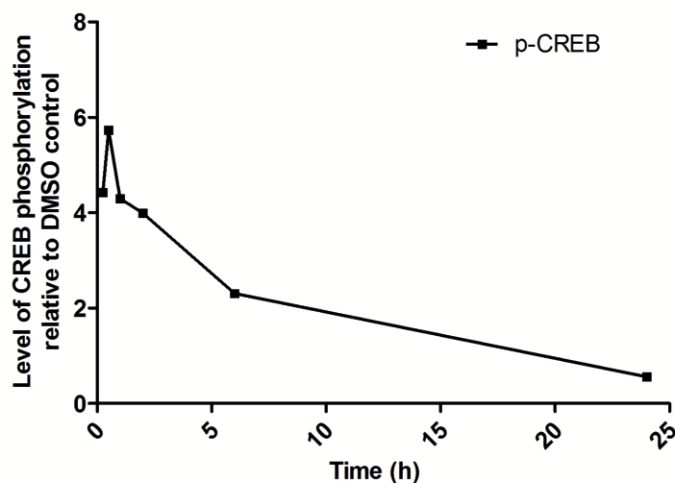

**Supplementary Figure 3B**

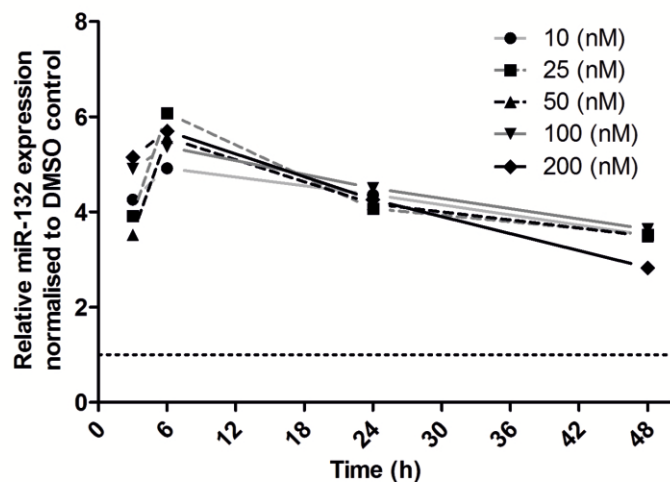

Supplement: Supplementary file 1 [file mmc1.pdf]
